# A synergistic effect of the triglyceride-glucose index and the residual SYNTAX score on the prediction of intermediate-term major adverse cardiac events in patients with type 2 diabetes mellitus undergoing percutaneous coronary intervention

**DOI:** 10.1186/s12933-022-01553-1

**Published:** 2022-06-24

**Authors:** Shiqiang Xiong, Qiang Chen, Zhen Zhang, Yingzhong Chen, Jun Hou, Caiyan Cui, Lianchao Cheng, Hong Su, Yu Long, Siqi Yang, Lingyao Qi, Xu Chen, Hanxiong Liu, Lin Cai

**Affiliations:** Department of Cardiology, the Third People’s Hospital of Chengdu, Affiliated Hospital of Southwest Jiaotong University, Chengdu, 610014 Sichuan China

**Keywords:** Triglyceride–glucose index, Residual SYNTAX score, Type 2 diabetes mellitus, Insulin resistance, Percutaneous coronary intervention, Major adverse cardiac events, Prognosis

## Abstract

**Background:**

The residual SYNTAX score (rSS), a quantitative measure of angiographic completeness of revascularization after percutaneous coronary intervention (PCI), and the triglyceride–glucose index (TyG index), a reliable surrogate marker of insulin resistance, have been regarded as independent predictors of major adverse cardiac events (MACEs) after PCI. Whether a combination of the rSS and the TyG index improves the predictive ability for MACEs in patients with type 2 diabetes mellitus (T2DM) undergoing PCI remains unknown.

**Methods:**

A total of 633 consecutive patients with T2DM who underwent PCI were included in the present analyses. Patients were stratified according to the optimal cutoff point value of the TyG index, or the rSS determined by receiver‑operating characteristic (ROC) curve analysis. The primary endpoint was the composite of MACEs, including all-cause death, nonfatal myocardial infarction, and unplanned repeat revascularization. Cumulative curves were calculated using the Kaplan–Meier method. Multivariate Cox regression was used to identify predictors of MACEs. The predictive value of the TyG index combined with the rSS was estimated by the area under the ROC curve, continuous net reclassification improvement (NRI) and integrated discrimination improvement (IDI).

**Results:**

During a median follow-up of 18.83 months, 99 patients developed MACEs, more frequently in the patients with a higher TyG index or rSS. Multivariate Cox hazards regression analysis revealed that both the TyG index and rSS were independent predictors of MACEs (hazard ratio 1.8004; 95% CI 1.2603–2.5718; P = 0.0012; 1.0423; 95% CI 1.0088–1.0769; P = 0.0129, respectively). Furthermore, Kaplan–Meier analysis demonstrated that both the TyG index and the rSS were significantly associated with an increased risk of MACEs (log-rank, all P < 0.01). The addition of the rSS and the TyG index to the baseline risk model had an incremental effect on the predictive value for MACE (increase in C-statistic value from 0.660 to 0.732; IDI 0.018; NRI 0.274; all P < 0.01).

**Conclusions:**

The TyG index predicts intermediate-term MACE after PCI in patients with T2DM independent of known cardiovascular risk factors. Adjustment of the rSS by the TyG index further improves the predictive ability for MACEs in patients with T2DM undergoing PCI.

**Supplementary Information:**

The online version contains supplementary material available at 10.1186/s12933-022-01553-1.

## Introduction

Coronary heart disease (CHD) is the leading cause of death worldwide, responsible for 8.9 million deaths and 16% of global mortality in 2019 [1]. Type 2 diabetes mellitus (T2DM) is a major risk factor for CHD. Insulin resistance (IR), a characteristic feature of T2DM, has been demonstrated to be significantly associated with the occurrence, progression, and prognosis of atherosclerotic cardiovascular disease [2]. Patients with T2DM usually have more extensive coronary atherosclerosis and a markedly increased incidence of adverse cardiovascular events and less favorable outcomes after coronary interventions [3]. The residual SYNTAX score (rSS), a quantitative measure of angiographic completeness of revascularization after percutaneous coronary intervention (PCI), has been validated as an independent predictor of adverse cardiovascular outcomes after PCI [4, 5]. Although it is well established that subjects with T2DM are more likely to have a poor prognosis after PCI, IR was not included as a variable in the rSS model.

The triglyceride-glucose (TyG) index, which is calculated from fasting glucose and triglycerides, has been proposed as a reliable surrogate indicator of IR [6]. The previous literature revealed that a high TyG index was related to increased coronary artery calcification progression and stenosis in asymptomatic adults [7, 8] and an increased risk of cardiovascular diseases in the general population [9–12]. Moreover, the TyG index showed a powerful ability for risk prediction after PCI in different cohorts, including patients with acute ST-elevation myocardial infarction (STEMI) [13], diabetic patients and nondiabetic patients with non-ST-segment elevation acute coronary syndrome (NSTE‑ACS) [14, 15], T2DM patients with ACS [16, 17], and nondiabetic CHD patients [18]. To the best of our knowledge, the prognostic value of the TyG index in patients with T2DM undergoing PCI has not been thoroughly investigated. Moreover, no relevant study has focused on whether the addition of the TyG index improves the predictive ability of the rSS in patients with T2DM undergoing PCI.

In the present study, we investigated the predictive value of the TyG index for adverse cardiovascular outcomes in patients with T2DM undergoing PCI and the potential incremental prognostic value of a combination of the TyG index with the rSS.

## Methods

### Study population

This study is a single-center, observational cohort study among patients with T2DM diagnosed with CHD and undergoing PCI at the Third People’s Hospital of Chengdu (Sichuan, China) between July 2018 and December 2020. The exclusion criteria were: (1) explicit or suspected type 1 diabetes mellitus; (2) incomplete clinical data and coronary angiography; (3) a history of coronary artery bypass grafting; (4) severe mechanical complications and valvular disease requiring cardiac surgery; (5) severe renal dysfunction (creatinine clearance < 15 ml/min); and (6) malignant tumor. Thirteen patients were also excluded because of missing follow-up data despite at least three separate attempts to contact them. Ultimately, a cohort of 633 patients who met the enrollment criteria were included in the present analyses. The study protocol was approved by the local research ethics committee and strictly adhered to the Declaration of Helsinki, with a waiver of informed consent. Personal information related to the identities of the patients was concealed.

### Data collection and definitions

Data on demographic, anthropometric, previous medical history, smoking, laboratory, medical and procedural information were extracted from the electronic medical record management system of the Third People’s Hospital of Chengdu.

Body mass index (BMI) was calculated as follows: BMI = weight (kg)/[height (m)]^2^. T2DM was defined according to one of the following criteria: (1) self-reported T2DM that was previously diagnosed by a physician or the use of antidiabetic medication (diet, oral agents, and/or insulin) before hospitalization; (2) the typical symptoms of T2DM with casual blood glucose ≥ 11.1 mmol/L, fasting blood glucose (FBG) ≥ 7.0 mmol/L, and/or 2-h blood glucose ≥ 11.1 mmol/L in the 75-g oral glucose tolerance test [19]. Hypertension was defined as a self-reported physician-diagnosed condition, currently receiving antihypertensive treatments, and/or systolic blood pressure (SBP) ≥ 140 mmHg and/or diastolic blood pressure (DBP) ≥ 90 mmHg at rest over three measurements [20]. A previous medical history of PCI, stroke, atrial fibrillation, and chronic obstructive pulmonary disease was obtained from self-reported information and then confirmed by relevant medical records. Acute myocardial infarction (AMI) was defined as the presence of acute myocardial injury detected by abnormal cardiac biomarkers with clinical evidence of acute myocardial ischemia [21].

Venous blood samples were taken after overnight fasting (> 8 h). Concentrations of cardiac troponin T (cTnT), brain natriuretic peptide (BNP), serum creatinine (Scr), FBG, hemoglobin A1c (HbA1c), triglycerides (TGs), total cholesterol (TC), high-density lipoprotein-C (HDL-C), and low-density lipoprotein-C (LDL-C), were determined by standard laboratory methods. The TyG index was calculated using the following formula: ln [fasting TG (mg/dL) × FBG (mg/dL)/2] [6]. Left ventricular ejection fraction (LVEF) was evaluated by the two-dimensional modified Simpson’s method.

### Baseline and residual SYNTAX score

In brief, the baseline SYNTAX score (bSS) was calculated from the preprocedural angiograms using the online calculation tool (http://syntaxscore.com/) by two experienced interventional cardiologists who were blinded to the baseline clinical characteristics and clinical outcomes. Each coronary lesion producing ≥ 50% in vessels ≥ 1.5 mm by visual estimation was regarded as a positive lesion and was included in the scoring [22, 23]. The rSS was calculated based on the remaining untreated obstructive coronary disease cases after treatment with PCI [4]. For patients undergoing staged PCI procedures (defined as a second planned PCI procedure after the initial intervention), the rSS after the last revascularization was adopted as the entry point for this study. In cases of disagreement, a third observer was consulted and the final decision was made by consensus. All data were assessed for quality and entered into a dedicated computer database.

### Follow-up and clinical endpoint definitions

Clinical follow-up was scheduled at 1, 3, 6 and 12 months, and then annually thereafter by clinical visit or telephone contact. Follow-up clinical events were investigated and recorded by professionals. The primary endpoint was defined as major adverse cardiac events (MACEs), a composite of all-cause death, non-fatal MI, and unplanned revascularization during follow-up. Secondary end points included all-cause death, cardiac death, nonfatal MI, nonfatal stroke, and unplanned revascularization. All-cause death referred to death attributed to cardiac or noncardiac causes. Death that could not be attributed to a noncardiac cause was considered cardiac death. Cardiac death was defined as death caused by MI, heart failure, sudden cardiac death, and cardiac procedures. Unplanned revascularization was defined as ischemia-driven target or nontarget revascularization [(PCI or coronary artery bypass grafting (CABG)] during the follow-up period. Stroke was defined as an ischemic or hemorrhagic stroke that occurred during the follow-up period (confirmed by imaging and diagnosed by a neurologist). All events were documented and verified by referring to relevant medical records if this information was available.

### Statistical analysis

Continuous variables are presented as the mean with standard deviation or median with interquartile range (IQR), and comparisons between two groups were examined by t-tests or Mann–Whitney U-tests, respectively. Categorical variables are described as frequencies and percentages, and comparisons between two groups were examined by the chi-square test. Receiver operating characteristic (ROC) curve analysis was performed to determine the optimal cutoff point value of the TyG index and the rSS for predicting the primary endpoint and to evaluate the predictive value of the TyG index and the rSS for the intermediate-term prognosis. The Kaplan–Meier method was performed to evaluate the rate of adverse cardiovascular events between groups according to the optimal cutoff point of the TyG index and the rSS, and discrepancies between groups were evaluated by log-rank tests. The predictive value of the variables for the intermediate-term prognosis of diabetic patients undergoing PCI was evaluated by univariate and multivariate Cox proportional hazards analyses. Moreover, the C-statistic, continuous net reclassification improvement (NRI), and integrated discrimination improvement (IDI) were determined to evaluate the discrimination capacity of the TyG index and the rSS to predict adverse cardiovascular events. A P value (two-tailed) < 0.05 was considered significant. All statistical analyses were performed with IBM SPSS Statistics version 26.0 (IBM Corporation, Chicago, IL, USA) and R Programming Language version 4.0.2 software (Vienna, Austria).

## Results

### Baseline characteristics of the total population

**Fig. 1:**
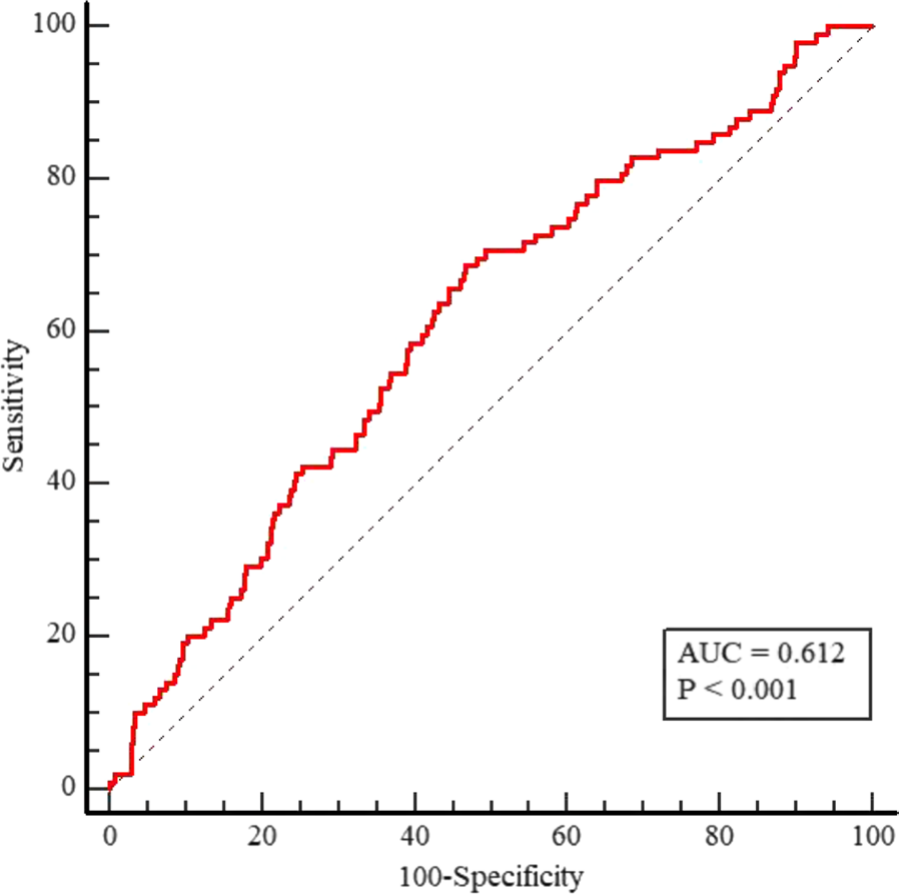
ROC curve analysis of the TyG index for MACEs. The receiver operating characteristic (ROC) curve of the triglyceride-glucose (TyG) index as a marker to predict MACEs in T2DM patients after PCI. The area under the ROC curve (AUC) of the TyG index for predicting the occurrence of MACEs in T2DM patients within 18 months after PCI was 0.612 (95% CI 0.573 to 0.650; P < 0.001). *T2DM* type 2 diabetes mellitus, *MACE* major adverse cardiac events, *PCI* percutaneous coronary intervention

Overall, 633 patients (68.02 ± 10.75 years, 32.8% female) who met the enrollment criteria and completed the follow-up were ultimately included in the present analyses. The baseline characteristics of the total population are summarized in Table 1. The levels of age and the TyG index were significantly higher in the patients with MACE. Patients who experienced MACE showed higher levels of BNP, Scr, and TG and lower levels of LVEF. Meanwhile, more patients were treated with diuretics and oral hypoglycemic agents in the MACE group. Compared with those without a primary endpoint, patients with MACE had lower rates of aspirin treatment. There were no significant differences between the groups in terms of diagnosis (chronic coronary syndrome, unstable angina, STEMI, and NSTEMI). Regarding coronary procedural information, patients with MACE exhibited higher rates of MVD, calcified lesions, and CTO and higher levels of bSS and rSS. Neither the number of stents nor the length of the stents differed between the two groups. The ROC analysis showed that the optimal cutoff value of the TyG index level for predicting MACEs in diabetic patients after PCI was 9.04 (sensitivity 68.69% and specificity 53.18%), with an area under the curve (AUC) of 0.612 (95% CI: 0.573 to 0.650, Fig. 1). The rate of MACE was higher in patients with a high TyG index (> 9.04).

**Table1:** Baseline characteristics of the patients stratified by the primary endpoint

Variable	All subjects (n = 633)	MACE-free (n = 534)	MACE (n = 99)	P value
Age, years	68.02 ± 10.75	67.52 ± 10.91	70.72 ± 9.41	0.007
Female	208 (32.8)	176 (33.0)	32 (32.3)	0.902
BMI, kg/m^2^	24.90 ± 3.10	24.99 ± 3.14	24.35 ± 2.85	0.057
Smoking, n (%)	284 (44.8)	238 (44.6)	46 (46.5)	0.728
Previous PCI, n (%)	68 (10.7)	54 (10.1)	14 (14.1)	0.234
COPD, n (%)	28 (4.4)	21 (3.9)	7 (7.1)	0.163
Hypertension, n (%)	477 (75.3)	401 (75.1)	76 (76.8)	0.723
AF, n (%)	52 (8.2)	40 (7.5)	12 (12.1)	0.123
Previous Stroke, n (%)	36 (5.6)	33 (6.2)	3 (3.0)	0.314
SBP, mmHg	133.66 ± 21.96	133.81 ± 21.66	132.86 ± 23.61	0.692
HR, bpm	78.78 ± 14.89	78.24 ± 14.76	81.72 ± 15.30	0.033
cTnT, pg/ml	30.41 (13.43, 484.75)	28.09 (13.17, 457.30)	56.68 (15.21, 608.90)	0.077
BNP, pg/ml	115.00 (42.10, 378.90)	107.65 (40.00, 340.77)	171.70 (50.30, 615.20)	0.008
Scr (umol/L)	77.90 (64.30, 97.55)	77.10 (64.00, 95.70)	82.40 (68.10, 116.60)	0.016
FBG (mmol/L)	8.38 ± 3.05	8.31 ± 3.03	8.78 ± 3.01	0.161
HbA1c	7.60 ± 1.72	7.58 ± 1.77	7.60 ± 1.71	0.936
TG (mmol/L)	1.56 ± 0.88	1.52 ± 0.83	1.77 ± 1.08	0.009
TC (mmol/L)	4.39 ± 1.27	4.38 ± 1.25	4.45 ± 1.38	0.579
HDL (mmol/L)	1.13 ± 0.29	2.65 ± 0.87	2.71 ± 0.99	0.472
LDL (mmol/L)	2.66 ± 0.89	1.12 ± 0.29	1.14 ± 0.29	0.529
LVEF	54.57 ± 9.71	55.16 ± 9.14	51.39 ± 11.89	< 0.001
AMI, n (%)	284 (44.8)	238 (44.6)	46 (46.5)	0.728
Diagnosis, n (%)				0.491
CCS	90 (14.2)	80 (15.0)	10 (10.1)	
UA	259 (40.9)	216 (40.4)	43 (43.4)	
NSTEMI	144 (22.7)	118 (22.1)	26 (26.3)	
STEMI	140 (22.1)	120 (22.5)	20 (20.2)	
Aspirin, n (%)	613 (96.8)	521 (97.6)	92 (92.9)	0.015
P2Y12 receptor inhibitor, n (%)	626 (98.8)	530 (99.3)	96 (97.0)	0.076
Statins, n (%)	618 (97.6)	523 (97.9)	95 (96.0)	0.234
β-blockers, n (%)	452 (71.4)	388 (72.7)	64 (64.6)	0.105
ACEI/ARB, n (%)	320 (50.5)	269 (50.4)	51 (51.5)	0.835
Diuretics, n (%)	139 (21.9)	106 (19.9)	33 (33.3)	0.003
Insulin, n (%)	167 (26.3)	133 (24.9)	34 (34.3)	0.05
Oral hypoglycemic agents, n (%)	450 (71.0)	388 (72.7)	62 (62.6)	0.043
MVD, n (%)	473 (74.7)	381 (71.3)	92 (92.9)	< 0.001
LM, n (%)	45 (7.1)	35 (6.6)	10 (10.1)	0.207
Calcified lesions, n (%)	119 (48.7)	92 (17.2)	27 (27.3)	0.019
Thrombosis, n (%)	58 (9.1)	48 (9.0)	10 (10.1)	0.725
Long lesion, n (%)	396 (62.5)	332 (62.2)	64 (64.6)	0.640
CTO, n (%)	139 (21.9)	101 (18.9)	38 (38.4)	< 0.001
Number of stents	1.54 ± 0.94	1.53 ± 0.95	1.59 ± 0.88	0.582
Length of stents, mm	40.82 ± 28.06	40.73 ± 28.30	41.29 ± 26.92	0.854
Tyg index	9.08 ± 0.56	9.04 ± 0.56	9.26 ± 0.55	< 0.001
TyG index ≤ 9.04	315 (49.7)	284 (53.2)	31 (31.3)	< 0.001
TyG index > 9.04	318 (50.2)	250 (46.8)	68 (68.7)	< 0.001
bSS	15.00 (9.00, 21.25)	14.50 (8.00, 20.50)	20.00 (14.00, 27.50)	< 0.001
rSS	4.00 (0.00, 9.00)	3.00 (0.00, 8.00)	8.00 (3.00, 13.00)	< 0.001

The patients were divided into two groups based on the primary endpoint. MACEs, major adverse cardiac events; BMI, body mass index; PCI, percutaneous coronary intervention; COPD, chronic obstructive pulmonary disease; AF, atrial fibrillation; SBP, systolic blood pressure; HR, heart rate; BNP, brain natriuretic peptide; Scr, serum creatinine; FBG, fasting blood glucose; TG, triglyceride; TC, total cholesterol; HDL, high density lipoprotein; LDL, low density lipoprotein; LVEF, left ventricular ejection fraction; AMI, acute myocardial infarction; CCS, chronic coronary syndrome; UA, unstable angina; STEMI, ST-segment elevation myocardial infarction; NSTEMI, non-ST-segment elevation myocardial infarction; ACEI/ARB, angiotensin converting enzyme inhibitor/angiotensin receptor blocker; MVD, multivessel disease; LM, left main disease; CTO, chronic total occlusion; TyG index, the triglyceride–glucose index; bSS, baseline SYNTAX score; rSS, residual SYNTAX score. Data are presented as mean ± SD, median (IQR) or n (%)

The baseline clinical and laboratory characteristics of the study patients according to the optimal cutoff point value of the TyG index to measure MACEs are presented in Table 2. Patients with a high TyG index (> 9.04) were more likely to be older, and had higher BMIs and heart rates. In terms of laboratory measurements, patients with a high TyG index had significantly higher levels of FBG, HbA1c, TG, TC, and LDL-C. Regarding the diagnosis, medications, angiographic findings and procedural results, there were no significant differences between the two groups.

**Table 2:** Baseline characteristics of the patients according to the optimal cut-off value of the TyG index

Variable	the Tyg index ≤ 9.04 (n = 315)	the Tyg index > 9.04 (n = 318)	P value
Age, years	69.31 ± 10.54	66.75 ± 10.81	0.003
Female	101 (32.1)	1.7 (33.6)	0.671
BMI, kg/m^2^	24.60 ± 2.93	25.19 ± 3.24	0.016
Smoking, n (%)	136 (43.2)	148 (46.5)	0.395
Previous PCI, n (%)	35 (11.1)	33 (10.4)	0.766
COPD, n (%)	15 (4.8)	13 (4.1)	0.680
Hypertension, n (%)	234 (74.3)	243 (76.4)	0.534
AF, n (%)	21 (6.7)	31 (9.7)	0.158
Previous Stroke, n (%)	18 (5.7)	18 (5.7)	0.977
SBP, mmHg	133.36 ± 22.64	133.96 ± 21.30	0.731
HR, bpm	77.43 ± 13.92	80.12 ± 15.69	0.023
cTnT, pg/ml	26.80 (13.50, 408.80)	38.84 (13.33, 604.93)	0.170
BNP, pg/ml	92.80 (39.20, 294.00)	130.00 (44.63, 462.81)	0.065
Scr (umol/L)	79.00 (67.00, 96.20)	76.10 (63.00, 100.98)	0.614
FBG (mmol/L)	7.08 ± 2.10	9.67 ± 3.29	< 0.001
HbA1c	7.26 ± 1.61	7.93 ± 1.76	< 0.001
TG (mmol/L)	1.11 ± 0.33	2.01 ± 1.02	< 0.001
TC (mmol/L)	4.10 ± 1.16	4.67 ± 1.31	< 0.001
HDL-C (mmol/L)	1.13 ± 0.30	1.12 ± 0.29	0.466
LDL-C (mmol/L)	2.47 ± 0.80	2.86 ± 0.93	< 0.001
LVEF	55.04 ± 9.50	55.11 ± 9.90	0.226
AMI, n (%)	130 (41.3)	154 (48.4)	0.070
Diagnosis,n (%)			0.150
CCS	52 (16.5)	38 (11.9)	
UA	133 (42.2)	126 (39.6)	
NSTEMI	70 (22.2)	74 (23.3)	
STEMI	60 (19.0)	80 (25.2)	
Aspirin, n (%)	309 (98.1)	304 (95.6)	0.072
P_2_Y_12_ receptor inhibitor, n (%)	313 (99.4)	313 (98.4)	0.259
Statins, n (%)	307 (97.5)	311 (97.8)	0.780
β-blockers, n (%)	214 (67.9)	238 (74.8)	0.055
ACEI/ARB, n (%)	151 (47.9)	169 (53.1)	0.190
Diuretics, n (%)	62 (19.7)	77 (24.2)	0.169
Insulin, n (%)	74 (23.5)	93 (29.2)	0.101
Oral hypoglycemic agents, n (%)	220 (69.8)	230 (72.3)	0.490
MVD, n (%)	227 (72.1)	246 (77.4)	0.125
LM, n (%)	24 (7.6)	21 (6.6)	0.619
Calcified lesions, n (%)	58 (18.4)	61 (19.2)	0.804
Thrombosis, n (%)	28 (8.9)	30 (9.4)	0.812
Long lesion, n (%)	193 (61.3)	203 (63.8)	0.504
CTO, n (%)	62 (19.7)	77 (24.2)	0.169
Number of stents	1.55 ± 0.94	1.53 ± 0.94	0.780
Length of stents, mm	40.54 ± 27.65	41.09 ± 28.50	0.805
Tyg index	8.65 ± 0.33	9.50 ± 0.40	< 0.001
bSS	15.00 (9.00,21.00)	15.75 (9.00,21.50)	0.458
rSS	4.00 (0.00,8.00)	4.25 (1.00,9.00)	0.327

The groups were stratified by the optimal cutoff value of the TyG index to measure MACEs, which was determined by receiver‑operating characteristic curve analysis. Data are presented as mean ± SD, median (IQR) or n (%)

*MACEs* major adverse cardiac events, *BMI* body mass index, *PCI* percutaneous coronary intervention, *COPD* chronic obstructive pulmonary disease, *AF* atrial fibrillation, *SBP* systolic blood pressure, *HR* heart rate, *BNP* brain natriuretic peptide, *Scr* serum creatinine, *FBG* fasting blood glucose, *TG* triglyceride, TC total cholesterol, *HDL* high density lipoprotein, *LDL* low density lipoprotein, *LVEF* left ventricular ejection fraction, *AMI* acute myocardial infarction, *CCS* chronic coronary syndrome, *UA* unstable angina, *STEMI* *ST*-segment elevation myocardial infarction, *NSTEMI* non-ST-segment elevation myocardial infarction, *ACEI*/*ARB* angiotensin converting enzyme inhibitor/angiotensin receptor blocker, *MVD* multivessel disease, *LM* left main disease, *CTO* chronic total occlusion, *TyG* index the triglyceride–glucose index, *bSS* baseline SYNTAX score, *rSS* residual SYNTAX score.

### Predictive value of the TyG index for the risk of adverse cardiovascular events

The median follow-up duration was 18.83 months (IQR, 14.55–22.88 months), and during the follow-up period, 35 (5.5%) all-cause deaths, 21 (3.3%) cardiac deaths, 20 (3.2%) nonfatal MIs, 22 (3.5%) nonfatal ischemic strokes, and 64 (10.1%) unplanned revascularizations were recorded (Table 3). Thus, a total of 99 (15.6%) MACEs were finally included in the present analysis. The incidence of MACE (21.4% vs. 9.8%) and unplanned revascularization (15.1% vs. 5.1%) in patients with a high TyG index (> 9.04) was significantly higher than in those with a lower TyG index (all P < 0.001), while the incidence of all-cause death, cardiac death, MI, and stroke was not significantly different between the two groups (Table 3).

**Table 3:** Comparison of intermediate-term adverse prognosis between two groups

Variable	the Tyg index ≤ 9.04 (n = 315)	the Tyg index > 9.04 (n = 318)	P value
MACE, n (%)	31 (9.8)	68 (21.4)	< 0.001
All-cause death, n (%)	13(4.1)	22 (6.9)	0.124
Cardiac death, n (%)	9 (2.9)	12 (3.8)	0.520
Myocardial infarction, n (%)	9 (2.9)	11 (3.5)	0.665
Unplanned revascularization, n (%)	16 (5.1)	48 (15.1)	< 0.001
Stroke, n (%)	14 (4.4)	8 (2.5)	0.180

Data are presented as n (%)

*MACEs* major adverse cardiac events, *TyG* index the triglyceride–glucose index.

**Fig. 2:**
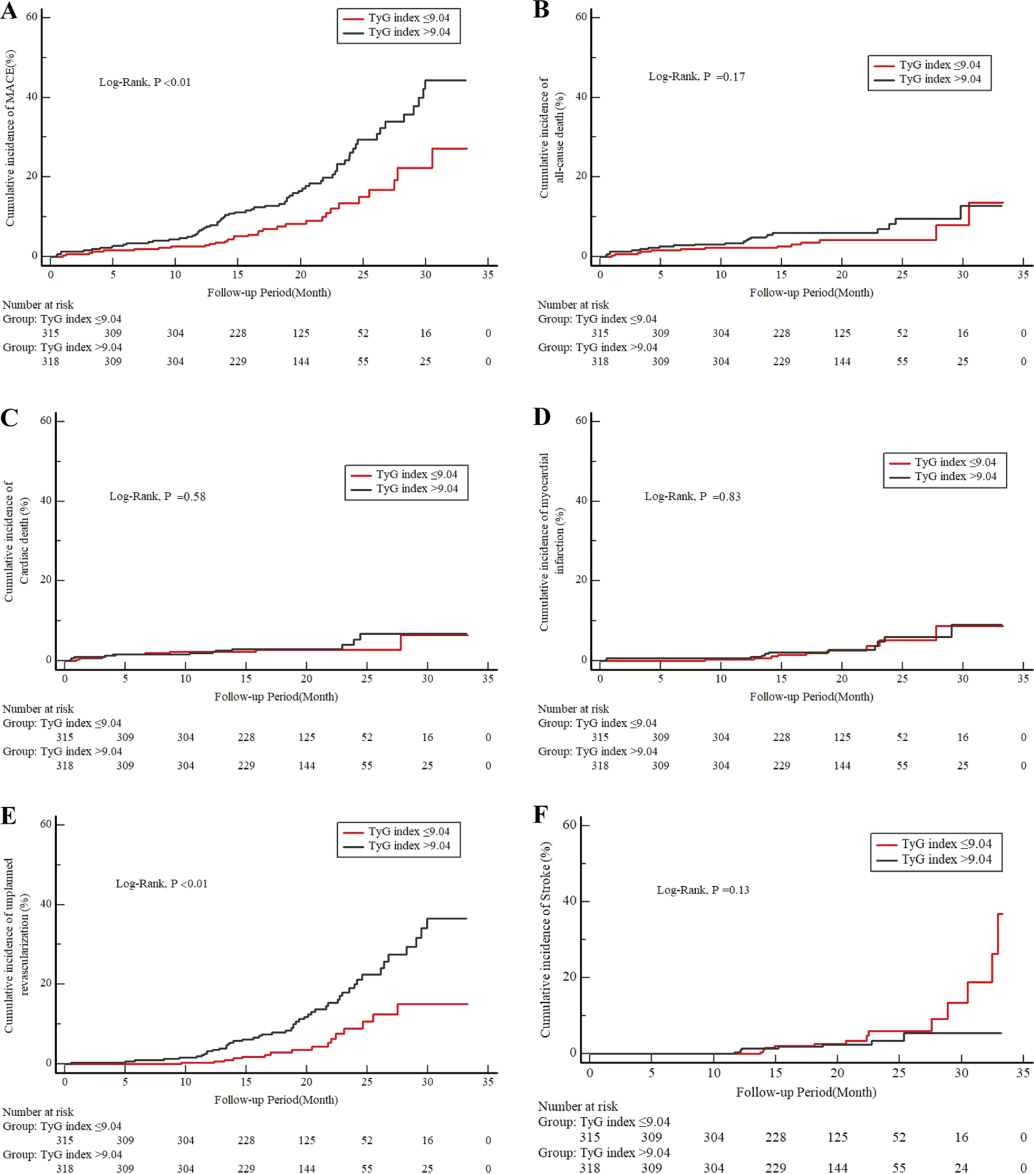
Cumulative incidence of endpoint events according to the optimal cutoff value of the TyG index. Kaplan–Meier curves for the incidence of the primary endpoint (**A**), all-cause death (**B**), cardiovascular death (**C**), nonfatal myocardial infarction (**D**), unplanned repeat revascularization (**E**), and nonfatal stroke (**F**) between the 2 study groups based on the TyG index. The groups were stratified by the optimal cutoff value of the TyG index determined by receiver‑operating characteristic curve analysis. TyG indicates triglyceride-glucose

The Kaplan–Meier analysis revealed that the cumulative incidence of the primary endpoint (MACE) was significantly higher in patients with a high TyG index (log-rank test, P < 0.01) (Fig. 2A). This difference was mainly driven by the increase in unplanned revascularization (log-rank test, P < 0.01) (Fig. 2E). In further analysis, we found that unplanned revascularization was mainly due to the progression of lesions rather than in-stent restenosis (Additional file 1: Figure S1). Meanwhile, the incidence of all-cause death (log-rank test, P = 0.17), cardiac death (log-rank test, P = 0.58), MI (log-rank test, P = 0.83), and stroke (log-rank test, P = 0.13) at follow-up were similar between the two groups (Fig. 2B–D, F).

Univariate and multivariate Cox proportional hazards regression analyses and predictors for MACEs are presented in Table 4. Univariate logistic regression showed that the TyG index, age, BMI, ACS, heart rate, BNP, Scr, bSS, rSS, LVEF, diuretics, and insulin were risk factors for MACEs in diabetic patients after PCI (all P < 0.05). After adjusting for BMI and other potential confounding factors, multivariate logistic regression showed that the TyG index, age, rSS, and LVEF were independent predictors of MACEs after PCI in patients with T2DM (all P < 0.05).

**Table 4:** Univariate and multivariate Cox regression analysis for predicting MACEs after PCI

Variables	Univariate analysis			Multivariate analysis		
	HR	95% CI	P value	HR	95% CI	P value
Age	1.0296	1.0100–1.0495	0.0029	1.0329	1.0119–1.0543	0.0020
Female	0.9596	0.6297–1.4624	0.8479			
BMI	0.9283	0.8696–0.9911	0.0258	0.9707	0.9069–1.0390	0.3910
ACS	2.0092	1.0415–3.8758	0.0374	1.8754	0.9594–3.6659	0.0659
Smoking	1.0485	0.7061–1.5569	0.8144			
Previous PCI	1.3494	0.7664–2.3759	0.2991			
Hypertension	1.1082	0.6949–1.7676	0.6661			
SBP	0.9963	0.9873–1.0053	0.4170			
Heart rate	1.0134	1.0007–1.0263	0.0389	1.0058	0.9925–1.0194	0.3928
BNP	1.0003	1.0001–1.0005	0.0056	1.0000	0.9997–1.0003	0.9703
cTnT	1.0000	1.0000–1.0001	0.5601			
Scr	1.0015	1.0006–1.0024	0.0014	1.0010	0.9999–1.0021	0.0782
HbA1c	0.9964	0.8913–1.1140	0.9499			
HDL	1.3501	0.6982–2.6107	0.3723			
LDL	1.0502	0.8396–1.3136	0.6680			
bSS	1.0543	1.0350–1.0741	< 0.001	1.0208	0.9940–1.0483	0.1295
rSS	1.0771	1.0520–1.1029	< 0.001	1.0406	1.0074–1.0750	0.0163
TyG index	1.6053	1.1586–2.2243	0.0044	1.7501	1.2239–2.5025	0.0022
LVEF	0.9680	0.9512–0.9850	< 0.001	0.9758	0.9531–0.9989	0.0406
β-blockers	0.7380	0.4887–1.1146	0.1487			
Diuretics	2.1210	1.3954–3.2238	< 0.001	1.2490	0.7550–2.0661	0.3866
ACEI/ARB	1.0856	0.7317–1.6105	0.6833			
Insulin	1.6609	1.0963–2.5163	0.0167	1.4817	0.9575–2.2929	0.0776

*MACEs* major adverse cardiac events, *PCI* percutaneous coronary intervention, *HR* hazard ratio, *CI* confidence interval, *BMI* body mass index, *ACS* acute coronary syndrome, *SBP* systolic blood pressure, *BNP* brain natriuretic peptide, *Scr* serum creatinine, *HDL* high density lipoprotein, *LDL* low density lipoprotein, *bSS* baseline SYNTAX score, *rSS* residual SYNTAX score, *TyG* index the triglyceride–glucose index, *LVEF* left ventricular ejection fraction, *ACEI*/*ARB* angiotensin converting enzyme inhibitor/angiotensin receptor blocker

### Diagnostic performance of the rSS for adverse cardiovascular events

**Fig. 3:**
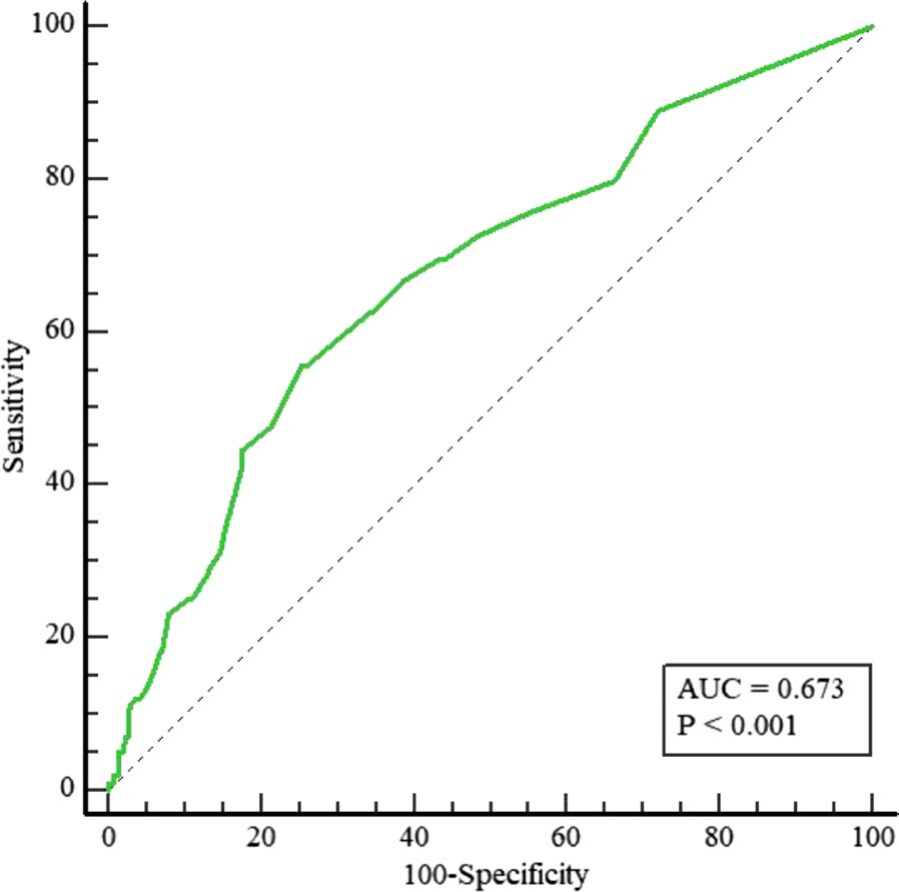
ROC curve analysis of the rSS for MACEs. The receiver operating characteristic (ROC) curve of the residual SYNTAX score (rSS) as a marker to predict MACEs in T2DM patients after PCI. The area under the ROC curve (AUC) of the rSS for predicting the occurrence of MACEs in T2DM patients within 18 months after PCI was 0.673 (95% CI 0.635 to 0.710, P < 0.001). T2DM, type 2 diabetes mellitus; MACE, major adverse cardiac events; PCI, percutaneous coronary intervention

**Fig. 4:**
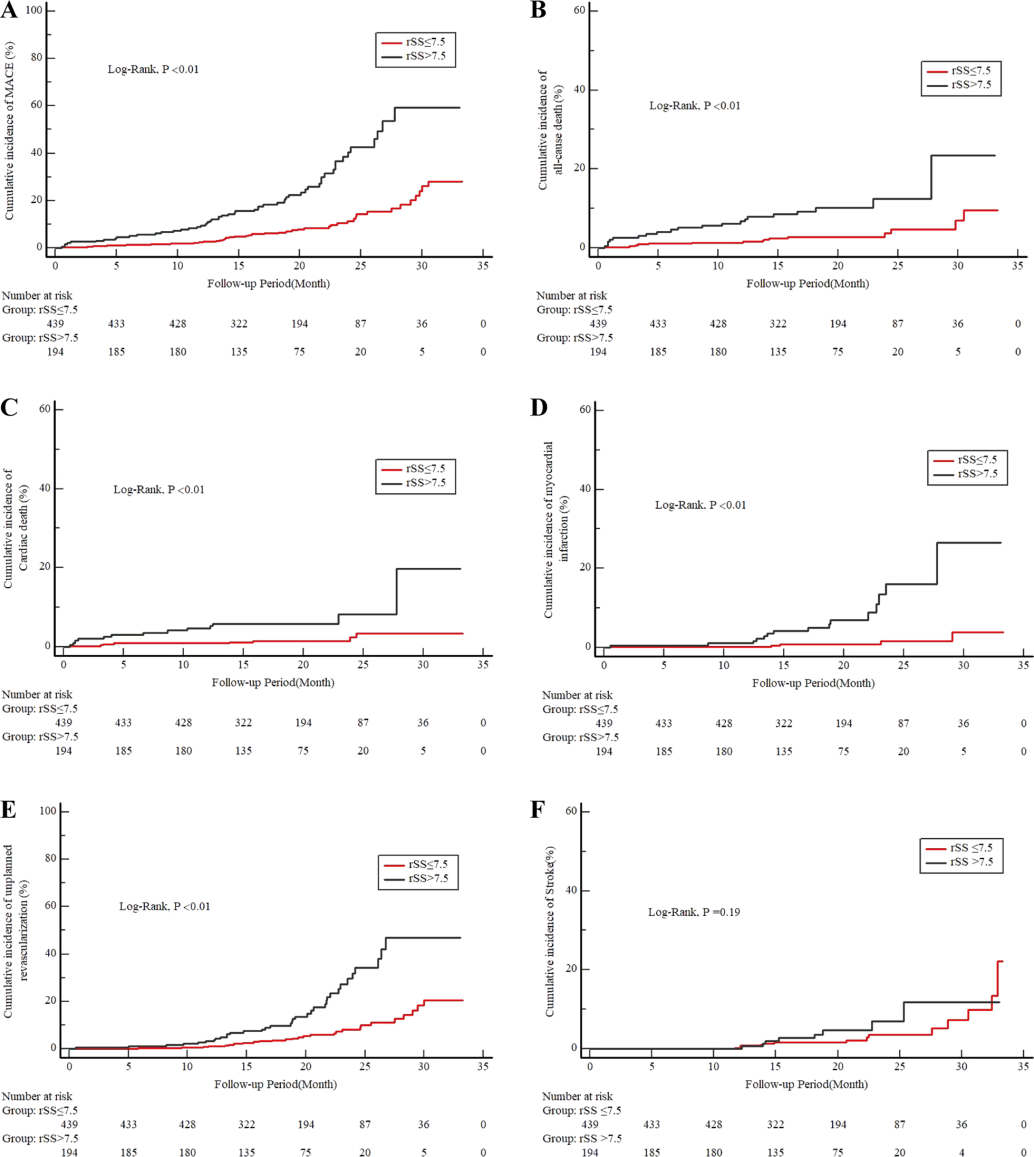
Cumulative incidence of endpoint events according to the optimal cutoff value of the rSS. Kaplan–Meier curves for the incidence of the primary endpoint (**A**), all-cause death (**B**), cardiovascular death (**C**), nonfatal myocardial infarction (**D**), unplanned repeat revascularization (**E**), and nonfatal stroke (**F**) between the 2 study groups based on the rSS. The groups were stratified by the optimal cutoff value of the rSS determined by receiver‑operating characteristic curve analysis. The rSS indicates the residual SYNTAX score

The diagnostic performance of the rSS for MACEs in diabetic patients after PCI was assessed by ROC analysis (Fig. 3). ROC analysis showed that the optimal cutoff value of the rSS for predicting MACEs was 7.5 (sensitivity 55.56% and specificity 74.53%), with an AUC of 0.673 (95% CI 0.635 to 0.710, P < 0.001). The Kaplan–Meier analysis further revealed that the cumulative incidences of MACEs, all-cause death, cardiac death, MI, and unplanned revascularization were significantly higher in patients with rSS > 7.5 at follow-up (all P < 0.01, Fig. 4A–E). The cumulative incidence of stroke was not significantly different between the two groups (log-rank test, P = 0.19; Fig. 4F). We demonstrated that the rSS had a potent predictive ability after PCI for patients with T2DM.

### Incremental effect of the TyG index on risk stratification for adverse cardiovascular events

**Fig. 5:**
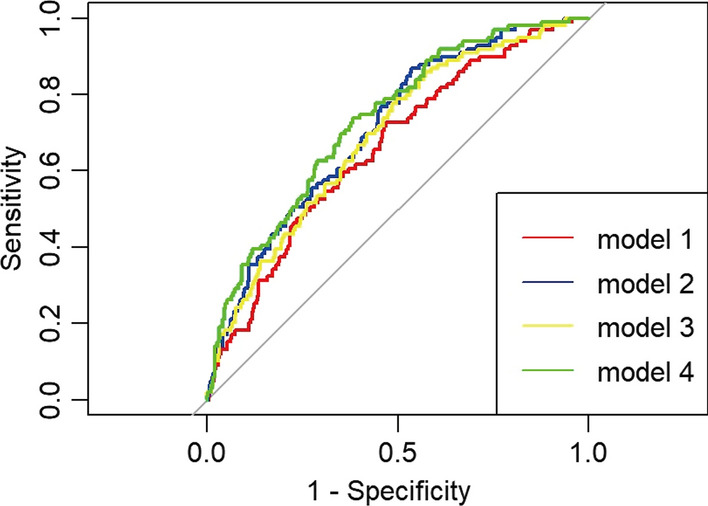
ROC curve analysis of the 4 models to predict MACEs after PCI in T2DM patients. The areas under the ROC curves of Model 1, Model 2, Model 3, and Model 4 for predicting the occurrence of MACEs in T2DM patients within 18 months after PCI were 0.660 (95% CI 0.622 to 0.697; P < 0.01), 0.710 (0.673 to 0.746; P < 0.01), 0.691 (0.653 to 0.726; P < 0.01), and 0.732 (0.696 to 0.766; P < 0.01), respectively. ROC, receiver operating characteristic; MACE, major adverse cardiac events; PCI, percutaneous coronary intervention; T2DM, type 2 diabetes mellitus

The synergistic effect of the TyG index and the rSS on the prediction of MACEs in patients with T2DM undergoing PCI is shown in Table 5 and Fig. 5. Compared with the baseline model of established risk factors (Model 1), the addition of the rSS (Model 2) had a significant increase in the C-statistic from 0.660 (95% CI 0.622–0.697) to 0.710 (95% CI 0.673–0.746) (P < 0.01) and a significant improvement in reclassification as assessed by the NRI (0.241, 95% CI 0.020–0.458, P = 0.03) and IDI (0.013, 95% CI 0.000–0.037, P = 0.04). The addition of the TyG index (Model 3) to the baseline model also resulted in a significant increase in the C-statistic from 0.660 (95% CI 0.622–0.697) to 0.691 (95% CI 0.653–0.726) (P < 0.01) and a significant improvement in reclassification as assessed by the NRI (0.268, 95% CI 0.060–0.422, P < 0.01) and IDI (0.017, 95% CI 0.003–0.054, P < 0.01). Moreover, the combination of the TyG index and the rSS (Model 4) had the strongest incremental effect for predicting MACEs in terms of the C-statistic from 0.660 (95% CI 0.622–0.697) to 0.732(95% CI 0.696 to 0.766), NRI (27.4% improvement, P = 0.01), and IDI (1.8% improvement, P < 0.01).

**Table 5:** Evaluation of Predictive Models for MACEs

Variables	NRI		IDI		C-Statistic	
	Index(95%CI)	P value	Index(95%CI)	P value	Index(95%CI)	P value
Model 1		Ref		Ref	0.660 (0.622–0.697)	< 0.01
Model 2	0.241 (0.020–0.458)	0.03	0.013 (0.000–0.037)	0.04	0.710 (0.673–0.746)	< 0.01
Model 3	0.268 (0.–0.422)	< 0.01	0.017 (0.003–0.054)	< 0.01	0.691 (0.653–0.726)	< 0.01
Model 4	0.274 (0.054–0.453)	0.01	0.018 (0.003–0.048)	< 0.01	0.732 (0.696–0.766)	< 0.01

Model 1 = baseline risk model, including age, heart rate, SBP, Serum creatinine, LVEF; Model 2 = Model 1 + rSS; Model 3 = Model 1 + Tyg index; Model 4 = Model 2 + Tyg index.

*MACEs* major adverse cardiac events, *SBP* systolic blood pressure, *LVEF* left ventricular ejection fraction, *NRI* net-reclassification index, *IDI* integrated discrimination improvement, *CI* confidence interval

## Discussion

In this study, we noticed that the TyG index was positively associated with adverse cardiovascular outcomes in patients with T2DM who underwent PCI. Even after adjustment for the potential confounding factors, the TyG index remained an independent predictor of MACEs. To the best of our knowledge, this study demonstrated, for the first time, that the addition of the TyG index improves the ability of models containing the rSS to predict intermediate-term adverse events in diabetic patients undergoing PCI. The results of our study suggest that adjustment of the rSS by the TyG index may optimize the risk stratification of recurrent cardiovascular risk in diabetic patients undergoing PCI.

The incidence of adverse cardiovascular events is significantly increased in diabetic patients after PCI compared with individuals without T2DM. More than 50% of diabetic patients die from cardiovascular events, especially CHD [24]. IR, a typical feature of T2DM, is usually characterized by increases in the FBG, fasting TG, fasting insulin, and visceral fat and decreased HDL-C, which is significantly associated with oxidative stress, inflammatory response, endothelial dysfunction, coagulation imbalance, and cardiovascular remodeling [25]. IR can induce imbalances in systemic glucose and lipid metabolism, leading to chronic hyperglycemia and dyslipidemia. These metabolic changes contribute to the pathogenesis and progression of CHD [25]. The TyG index, which is a simple, cost-effective, and reliable surrogate marker of IR, has been demonstrated to be well correlated with the results of hyperinsulinemic–euglycemic clamp and HOMA-IR in both healthy and diabetic individuals [26]. Therefore, it is of great clinical significance to determine the role of the TyG index in risk stratification and prognosis prediction in patients with T2DM undergoing PCI.

Our study population represents a cohort of patients with T2DM and angiographically proven CHD in which the association between the TyG index and intermediate-term prognosis has been investigated. We demonstrated that a higher TyG index was independently associated with worse cardiovascular outcomes, mainly driven by the increase in unplanned revascularization. Patients with unplanned revascularization have been demonstrated to have substantially higher risks of subsequent rehospitalization than subjects without such events [27]. The progression of lesions was the main factor contributing to unplanned revascularization in the present cohort, rather than in-stent restenosis. Our findings are different from those of a recent study demonstrating that an elevated TyG index was independently and positively associated with in-stent restenosis in patients with ACS after PCI with drug-eluting stents [28]. Differences in subject selection may contribute to the discrepancy of these results.

It is of great importance for physicians to manage patients according to their risk stratification. The rSS, which is an objective, anatomical index calculated from the degree and complexity of residual stenosis after PCI, has been demonstrated to be an independent predictor of adverse cardiovascular events after PCI in different population cohorts [4, 29–31]. The incidence of adverse cardiovascular events was significantly higher after PCI in patients with rSS > 8 than in those with rSS ≤ 8 [4, 29–31]. We found that the incidence of MACEs, all-cause death, cardiac death, MI and unplanned revascularization was higher after PCI in T2DM patients with rSS > 7.5 than in those with rSS ≤ 7.5. Our findings also demonstrated the value of prognosis prediction and risk stratification by rSS in patients with T2DM undergoing PCI. However, rSS does not include any cardiovascular metabolic risk factors in its model, and the clinical use of rSS has some limitations. By adding rSS to established risk factors of MACEs, we found a significant improvement in risk prediction in terms of the C-statistic value, NRI and IDI. Furthermore, the combination of the TyG index and the rSS produced a stronger predictive value, which improved the model discrimination and risk reclassification abilities. Our results implied that clinicians could apply the TyG index in combination with the rSS to identify higher-risk diabetic patients after PCI and thus apply a more targeted prevention or aggressive treatment to improve their clinical outcomes.

Treatment strategies are aimed not only at resolving the presenting pathology but also at reducing the risk of poor cardiovascular outcomes. Recent cardiovascular outcome trials suggest that treatment of IR is a promising intervention for diabetic patients at risk for adverse cardiovascular events [32]. Pioglitazone, which is a potent insulin sensitizer, has been demonstrated to blunt atherosclerotic progression (PERISCOPE and Chicago) and reduce the rate of cardiovascular events in large randomized prospective cardiovascular outcome trials (IRIS and PROactive) [33–36]. Whether aggressive treatment with IR can improve the prognosis of diabetic patients who have a higher rSS after PCI needs further comprehensive investigation in clinical practice.

### Study limitations

First, the present study was a retrospective analysis derived from a single-center with a relatively small sample size. These findings need to be verified by prospective, multicenter and large cohort studies. Second, the levels of FPG and triglycerides were assessed at baseline, which could be affected by the use of lipid-lowering drugs and antidiabetic agents and changed by follow-up; therefore, it is unknown whether the fluctuations in the TyG index impact its predictive value for diabetic patients undergoing PCI, which deserves further investigation. Additionally, it is of great significance to identify patients with subclinical IR and investigate the impact of IR on cardiovascular outcomes in future studies. Third, the relatively small number of hard endpoints observed in our cohort made it difficult to make any conclusions about the relationship between the TyG index and these individual events, longer follow-up time and/or larger cohort studies may help to illustrate these issues. Finally, this study was focused on Chinese participants, so conclusions for other ethnic groups require further study.

## Conclusion

A higher TyG index was independently associated with an increased risk of MACEs after PCI in patients with T2DM. A combination of the TyG index and the rSS has incremental prognostic value for the prediction of MACEs. These findings suggest that physicians may apply the TyG index in combination with the rSS to identify diabetic patients with high residual risk after PCI, and thus they can be subjected to targeted prevention or aggressive treatment to improve their clinical outcomes.

## Supplementary Information

**Additional file 1: Figure S1. **Reasons contribute to unplanned revascularization

## Data Availability

The datasets used and/or analyzed in the study are available from the corresponding author upon reasonable request.

## Abbreviations

MACEs: Major adverse cardiac events
T2DM: Type 2 diabetes mellitus
IR: Insulin resistance
BMI: Body mass index
PCI: Percutaneous coronary intervention
COPD: Chronic obstructive pulmonary disease
AF: Atrial fibrillation
SBP: Systolic blood pressure
HR: Heart rate
BNP: Brain natriuretic peptide
Scr: Serum creatinine
FBG: Fasting blood glucose
TG: Triglyceride
TC: Total cholesterol
HDL: High density lipoprotein
LDL: Low density lipoprotein
LVEF: Left ventricular ejection fraction
AMI: Acute myocardial infarction
ACEI/ARB: Angiotensin converting enzyme inhibitor/angiotensin receptor blocker
MVD: Multivessel disease
LM: Left main disease
CTO: Chronic total occlusion
TyG index: The triglyceride–glucose index
bSS: Baseline SYNTAX score
rSS: Residual SYNTAX score
