# Supplementary material for: A synergistic effect of the triglyceride-glucose index and the residual SYNTAX score on the prediction of intermediate-term major adverse cardiac events in patients with type 2 diabetes mellitus undergoing percutaneous coronary intervention
Source: Cardiovasc Diabetol. 2022 Jun 24;21:115. doi: 10.1186/s12933-022-01553-1 (PMC9233313; doi:10.1186/s12933-022-01553-1)
Supplement: Supplementary file 1 — Additional file 1: Figure S1. Reasons contribute to unplanned revascularization [file 12933_2022_1553_MOESM1_ESM.docx]

**
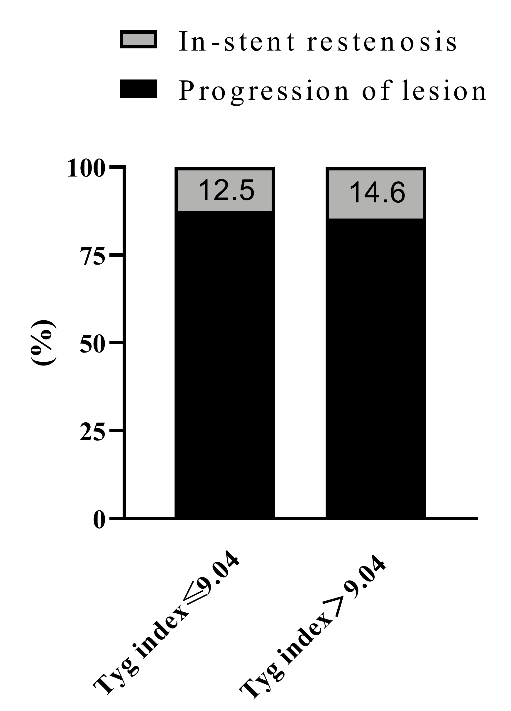
**

**Figure S1 Reasons contribute to unplanned revascularization**

In patients with Tyg index≤ 9.04, a total of 16 patients received unplanned revascularization [2 patients (12.5%) due to in-stent restenosis, and 14 patients (87.5%) due to progression of lesion]. In patients with Tyg index> 9.04, a total of 48 patients received unplanned revascularization [7 patients (14.6%) due to in-stent restenosis, and 41 patients (85.4%) due to progression of lesion].
